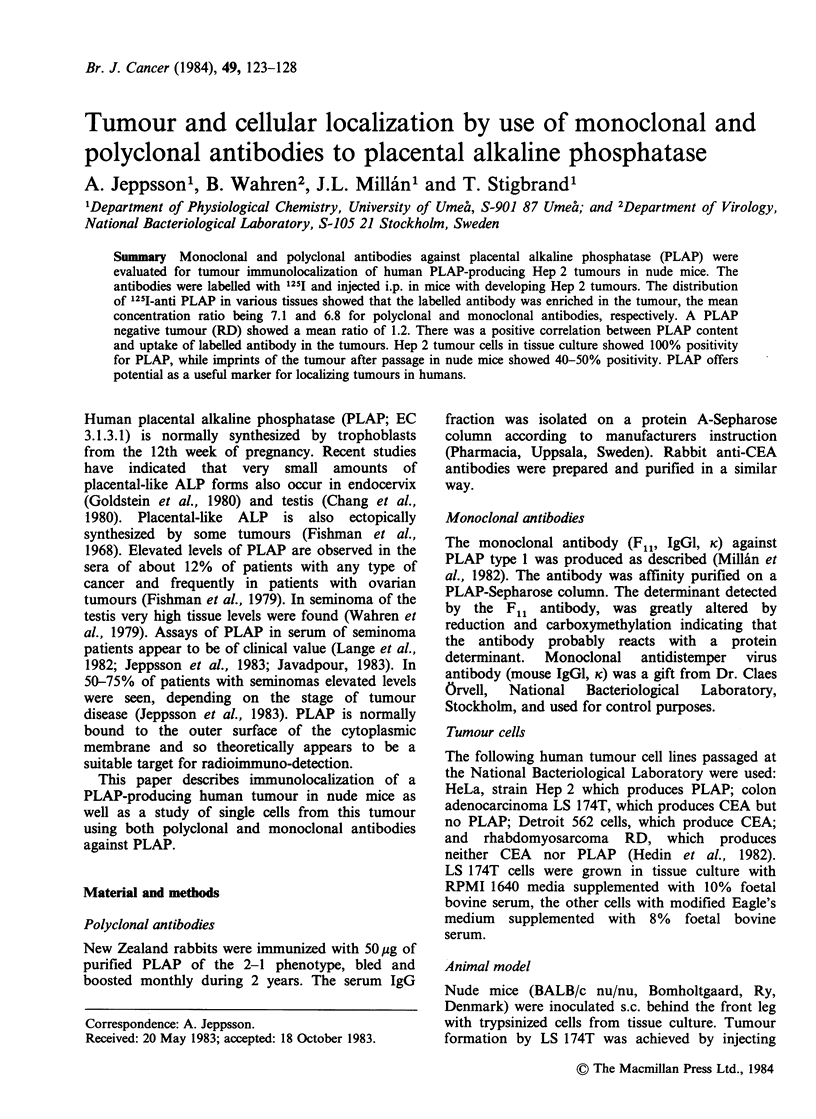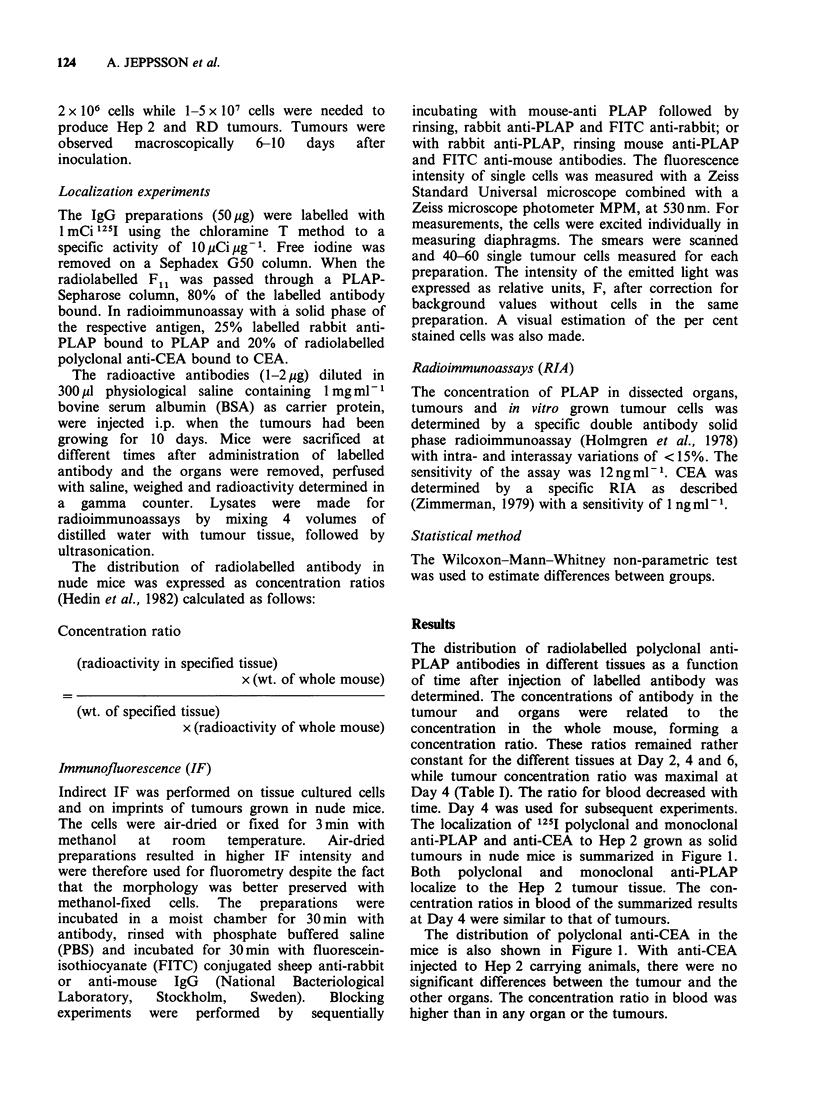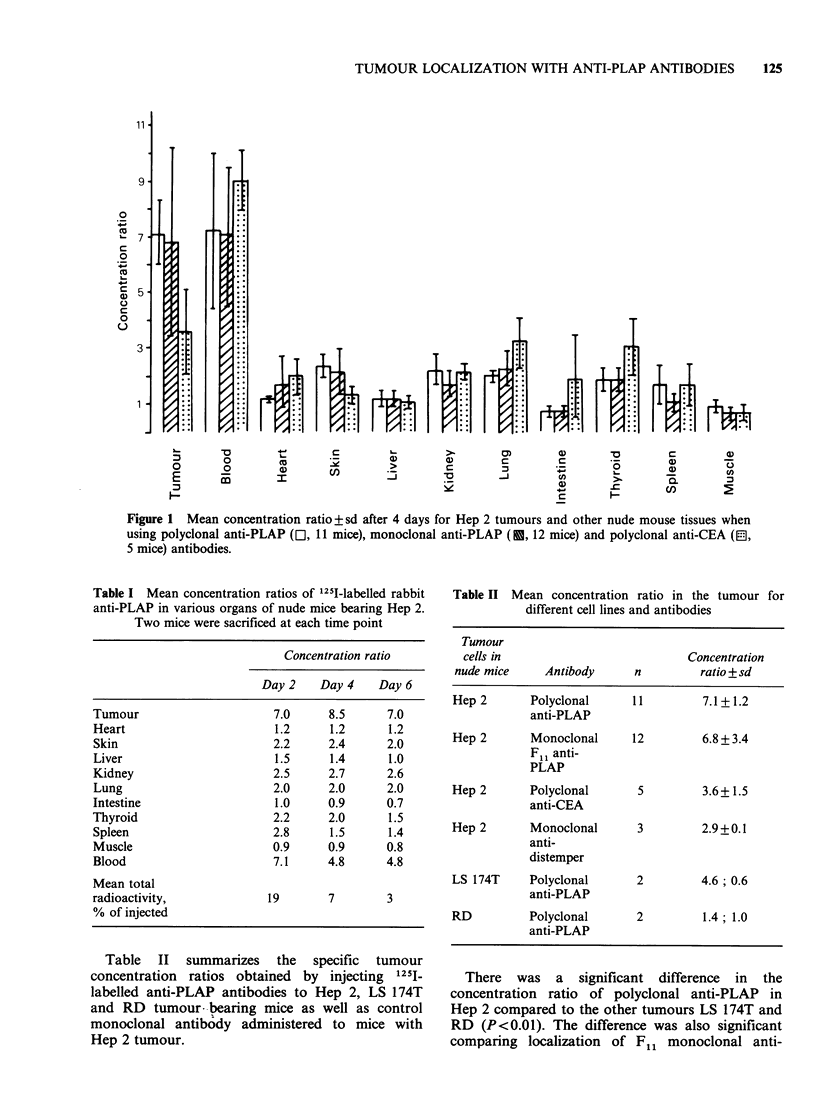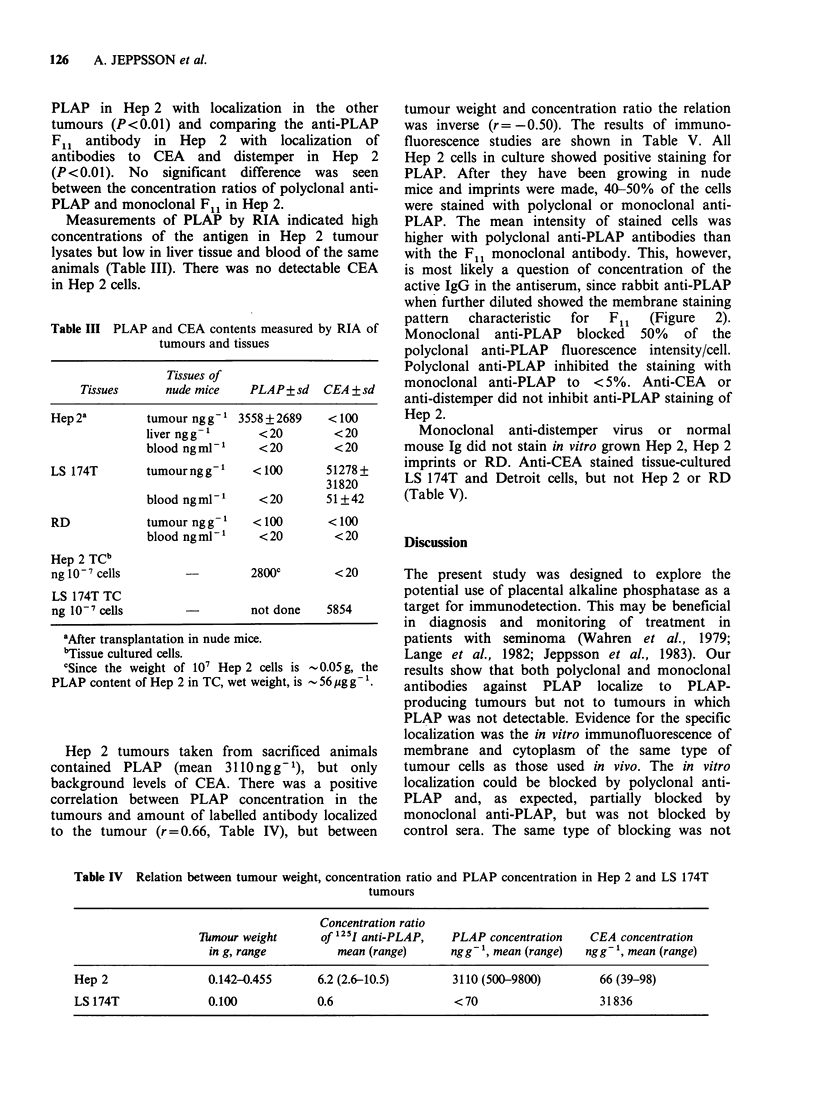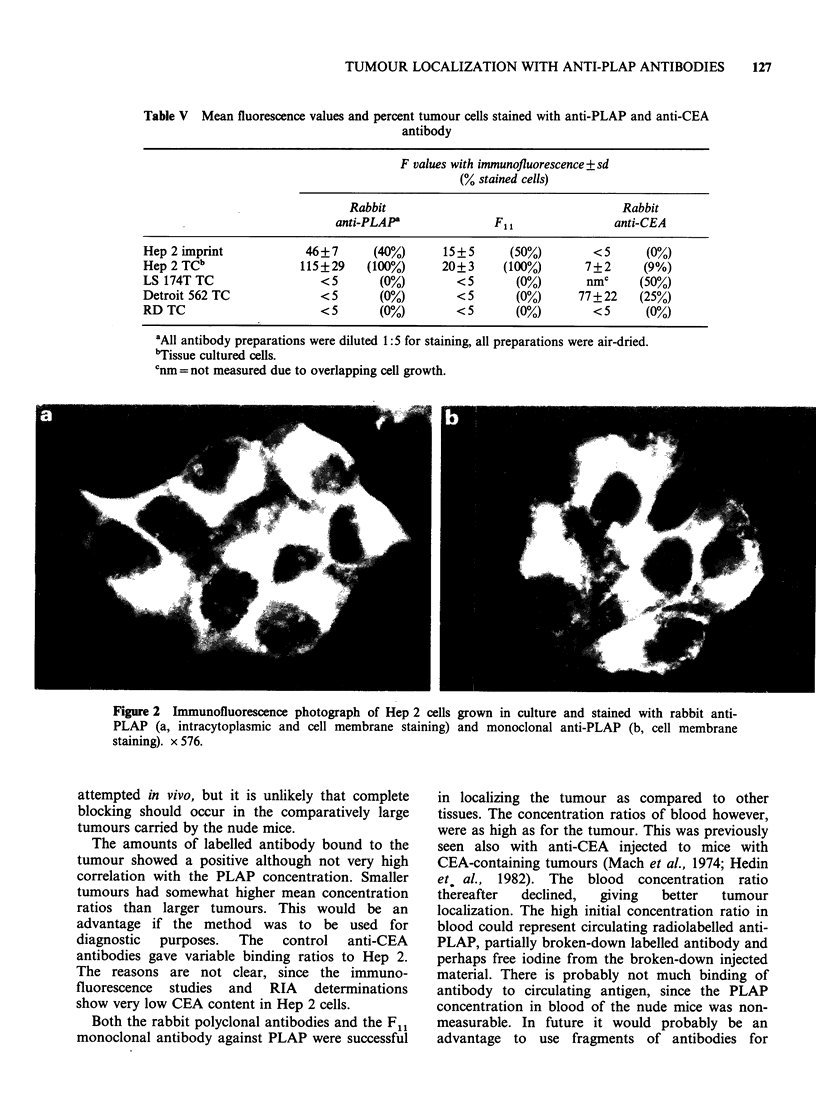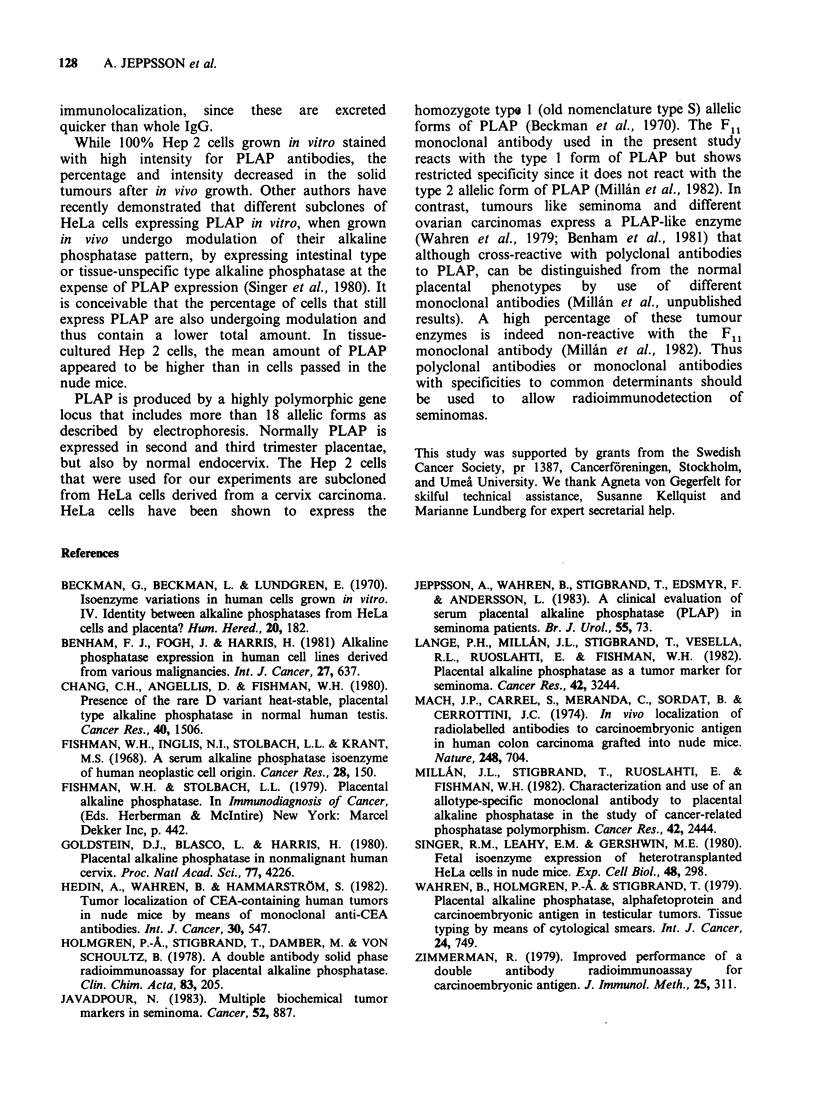# Tumour and cellular localization by use of monoclonal and polyclonal antibodies to placental alkaline phosphatase.

**DOI:** 10.1038/bjc.1984.23

**Published:** 1984-02

**Authors:** A. Jeppsson, B. Wahren, J. L. Millán, T. Stigbrand

## Abstract

**Images:**


*Br. J. Cancer* (1984), **49**, 123–128

### Tumour and cellular localization by use of monoclonal and polyclonal antibodies to placental alkaline phosphatase

A. Jeppsson[1], B. Wahren[2], J.L. Millán[1] and T. Stigbrand[1]

[1]*Department of Physiological Chemistry, University of Umeå, S-901 87 Umeå; and* [2]*Department of Virology, National Bacteriological Laboratory, S-105 21 Stockholm, Sweden*

**Summary** Monoclonal and polyclonal antibodies against placental alkaline phosphatase (PLAP) were evaluated for tumour immunolocalization of human PLAP-producing Hep 2 tumours in nude mice. The antibodies were labelled with $^{125}$I and injected i.p. in mice with developing Hep 2 tumours. The distribution of $^{125}$I-anti PLAP in various tissues showed that the labelled antibody was enriched in the tumour, the mean concentration ratio being 7.1 and 6.8 for polyclonal and monoclonal antibodies, respectively. A PLAP negative tumour (RD) showed a mean ratio of 1.2. There was a positive correlation between PLAP content and uptake of labelled antibody in the tumours. Hep 2 tumour cells in tissue culture showed 100% positivity for PLAP, while imprints of the tumour after passage in nude mice showed 40–50% positivity. PLAP offers potential as a useful marker for localizing tumours in humans.

Human placental alkaline phosphatase (PLAP; EC 3.1.3.1) is normally synthesized by trophoblasts from the 12th week of pregnancy. Recent studies have indicated that very small amounts of placental-like ALP forms also occur in endocervix (Goldstein *et al.*, 1980) and testis (Chang *et al.*, 1980). Placental-like ALP is also ectopically synthesized by some tumours (Fishman *et al.*, 1968). Elevated levels of PLAP are observed in the sera of about 12% of patients with any type of cancer and frequently in patients with ovarian tumours (Fishman *et al.*, 1979). In seminoma of the testis very high tissue levels were found (Wahren *et al.*, 1979). Assays of PLAP in serum of seminoma patients appear to be of clinical value (Lange *et al.*, 1982; Jeppsson *et al.*, 1983; Javadpour, 1983). In 50–75% of patients with seminomas elevated levels were seen, depending on the stage of tumour disease (Jeppsson *et al.*, 1983). PLAP is normally bound to the outer surface of the cytoplasmic membrane and so theoretically appears to be a suitable target for radioimmuno-detection.

This paper describes immunolocalization of a PLAP-producing human tumour in nude mice as well as a study of single cells from this tumour using both polyclonal and monoclonal antibodies against PLAP.

### Material and methods

### *Polyclonal antibodies*

New Zealand rabbits were immunized with 50 $\mu$g of purified PLAP of the 2–1 phenotype, bled and boosted monthly during 2 years. The serum IgG fraction was isolated on a protein A-Sepharose column according to manufacturers instruction (Pharmacia, Uppsala, Sweden). Rabbit anti-CEA antibodies were prepared and purified in a similar way.

### *Monoclonal antibodies*

The monoclonal antibody ($F_{11}$, IgGl, $\kappa$) against PLAP type 1 was produced as described (Millán *et al.*, 1982). The antibody was affinity purified on a PLAP-Sepharose column. The determinant detected by the $F_{11}$ antibody, was greatly altered by reduction and carboxymethylation indicating that the antibody probably reacts with a protein determinant. Monoclonal antidistemper virus antibody (mouse IgGl, $\kappa$) was a gift from Dr. Claes Örvell, National Bacteriological Laboratory, Stockholm, and used for control purposes.

### *Tumour cells*

The following human tumour cell lines passaged at the National Bacteriological Laboratory were used: HeLa, strain Hep 2 which produces PLAP; colon adenocarcinoma LS 174T, which produces CEA but no PLAP; Detroit 562 cells, which produce CEA; and rhabdomyosarcoma RD, which produces neither CEA nor PLAP (Hedin *et al.*, 1982). LS 174T cells were grown in tissue culture with RPMI 1640 media supplemented with 10% foetal bovine serum, the other cells with modified Eagle's medium supplemented with 8% foetal bovine serum.

### *Animal model*

Nude mice (BALB/c nu/nu, Bomholtgaard, Ry, Denmark) were inoculated s.c. behind the front leg with trypsinized cells from tissue culture. Tumour formation by LS 174T was achieved by injecting

Correspondence: A. Jeppsson.
Received: 20 May 1983; accepted: 18 October 1983.

© The Macmillan Press Ltd., 1984

$2 \times 10^6$ cells while $1–5 \times 10^7$ cells were needed to produce Hep 2 and RD tumours. Tumours were observed macroscopically 6–10 days after inoculation.

*Localization experiments*

The IgG preparations (50 $\mu$g) were labelled with 1 mCi $^{125}$I using the chloramine T method to a specific activity of 10 $\mu$Ci $\mu g^{-1}$. Free iodine was removed on a Sephadex G50 column. When the radiolabelled $F_{11}$ was passed through a PLAP-Sepharose column, 80% of the labelled antibody bound. In radioimmunoassay with a solid phase of the respective antigen, 25% labelled rabbit anti-PLAP bound to PLAP and 20% of radiolabelled polyclonal anti-CEA bound to CEA.

The radioactive antibodies (1–2 $\mu$g) diluted in 300 $\mu$l physiological saline containing 1 mg ml$^{-1}$ bovine serum albumin (BSA) as carrier protein, were injected i.p. when the tumours had been growing for 10 days. Mice were sacrificed at different times after administration of labelled antibody and the organs were removed, perfused with saline, weighed and radioactivity determined in a gamma counter. Lysates were made for radioimmunoassays by mixing 4 volumes of distilled water with tumour tissue, followed by ultrasonication.

The distribution of radiolabelled antibody in nude mice was expressed as concentration ratios (Hedin *et al.*, 1982) calculated as follows:

Concentration ratio

$$= \frac{(\text{radioactivity in specified tissue}) \times (\text{wt. of whole mouse})}{(\text{wt. of specified tissue}) \times (\text{radioactivity of whole mouse})}$$

*Immunofluorescence (IF)*

Indirect IF was performed on tissue cultured cells and on imprints of tumours grown in nude mice. The cells were air-dried or fixed for 3 min with methanol at room temperature. Air-dried preparations resulted in higher IF intensity and were therefore used for fluorometry despite the fact that the morphology was better preserved with methanol-fixed cells. The preparations were incubated in a moist chamber for 30 min with antibody, rinsed with phosphate buffered saline (PBS) and incubated for 30 min with fluorescein-isothiocyanate (FITC) conjugated sheep anti-rabbit or anti-mouse IgG (National Bacteriological Laboratory, Stockholm, Sweden). Blocking experiments were performed by sequentially incubating with mouse-anti PLAP followed by rinsing, rabbit anti-PLAP and FITC anti-rabbit; or with rabbit anti-PLAP, rinsing mouse anti-PLAP and FITC anti-mouse antibodies. The fluorescence intensity of single cells was measured with a Zeiss Standard Universal microscope combined with a Zeiss microscope photometer MPM, at 530 nm. For measurements, the cells were excited individually in measuring diaphragms. The smears were scanned and 40–60 single tumour cells measured for each preparation. The intensity of the emitted light was expressed as relative units, F, after correction for background values without cells in the same preparation. A visual estimation of the per cent stained cells was also made.

*Radioimmunoassays (RIA)*

The concentration of PLAP in dissected organs, tumours and *in vitro* grown tumour cells was determined by a specific double antibody solid phase radioimmunoassay (Holmgren *et al.*, 1978) with intra- and interassay variations of $<15\%$. The sensitivity of the assay was 12 ng ml$^{-1}$. CEA was determined by a specific RIA as described (Zimmerman, 1979) with a sensitivity of 1 ng ml$^{-1}$.

*Statistical method*

The Wilcoxon–Mann–Whitney non-parametric test was used to estimate differences between groups.

### Results

The distribution of radiolabelled polyclonal anti-PLAP antibodies in different tissues as a function of time after injection of labelled antibody was determined. The concentrations of antibody in the tumour and organs were related to the concentration in the whole mouse, forming a concentration ratio. These ratios remained rather constant for the different tissues at Day 2, 4 and 6, while tumour concentration ratio was maximal at Day 4 (Table I). The ratio for blood decreased with time. Day 4 was used for subsequent experiments. The localization of $^{125}$I polyclonal and monoclonal anti-PLAP and anti-CEA to Hep 2 grown as solid tumours in nude mice is summarized in Figure 1. Both polyclonal and monoclonal anti-PLAP localize to the Hep 2 tumour tissue. The concentration ratios in blood of the summarized results at Day 4 were similar to that of tumours.

The distribution of polyclonal anti-CEA in the mice is also shown in Figure 1. With anti-CEA injected to Hep 2 carrying animals, there were no significant differences between the tumour and the other organs. The concentration ratio in blood was higher than in any organ or the tumours.

**Figure 1** Mean concentration ratio ± sd after 4 days for Hep 2 tumours and other nude mouse tissues when using polyclonal anti-PLAP (□, 11 mice), monoclonal anti-PLAP (▨, 12 mice) and polyclonal anti-CEA (▤, 5 mice) antibodies.

**Table I** Mean concentration ratios of $^{125}$I-labelled rabbit anti-PLAP in various organs of nude mice bearing Hep 2. Two mice were sacrificed at each time point

| | *Concentration ratio* | | |
|---|---|---|---|
| | *Day 2* | *Day 4* | *Day 6* |
| Tumour | 7.0 | 8.5 | 7.0 |
| Heart | 1.2 | 1.2 | 1.2 |
| Skin | 2.2 | 2.4 | 2.0 |
| Liver | 1.5 | 1.4 | 1.0 |
| Kidney | 2.5 | 2.7 | 2.6 |
| Lung | 2.0 | 2.0 | 2.0 |
| Intestine | 1.0 | 0.9 | 0.7 |
| Thyroid | 2.2 | 2.0 | 1.5 |
| Spleen | 2.8 | 1.5 | 1.4 |
| Muscle | 0.9 | 0.9 | 0.8 |
| Blood | 7.1 | 4.8 | 4.8 |
| Mean total radioactivity, % of injected | 19 | 7 | 3 |

Table II summarizes the specific tumour concentration ratios obtained by injecting $^{125}$I-labelled anti-PLAP antibodies to Hep 2, LS 174T and RD tumour bearing mice as well as control monoclonal antibody administered to mice with Hep 2 tumour.

**Table II** Mean concentration ratio in the tumour for different cell lines and antibodies

| *Tumour cells in nude mice* | *Antibody* | *n* | *Concentration ratio ± sd* |
|---|---|---|---|
| Hep 2 | Polyclonal anti-PLAP | 11 | 7.1 ± 1.2 |
| Hep 2 | Monoclonal $F_{11}$ anti-PLAP | 12 | 6.8 ± 3.4 |
| Hep 2 | Polyclonal anti-CEA | 5 | 3.6 ± 1.5 |
| Hep 2 | Monoclonal anti-distemper | 3 | 2.9 ± 0.1 |
| LS 174T | Polyclonal anti-PLAP | 2 | 4.6 ; 0.6 |
| RD | Polyclonal anti-PLAP | 2 | 1.4 ; 1.0 |

There was a significant difference in the concentration ratio of polyclonal anti-PLAP in Hep 2 compared to the other tumours LS 174T and RD ($P<0.01$). The difference was also significant comparing localization of $F_{11}$ monoclonal anti-

PLAP in Hep 2 with localization in the other tumours ($P<0.01$) and comparing the anti-PLAP $F_{11}$ antibody in Hep 2 with localization of antibodies to CEA and distemper in Hep 2 ($P<0.01$). No significant difference was seen between the concentration ratios of polyclonal anti-PLAP and monoclonal $F_{11}$ in Hep 2.

Measurements of PLAP by RIA indicated high concentrations of the antigen in Hep 2 tumour lysates but low in liver tissue and blood of the same animals (Table III). There was no detectable CEA in Hep 2 cells.

**Table III** PLAP and CEA contents measured by RIA of tumours and tissues

| *Tissues* | *Tissues of nude mice* | *PLAP ± sd* | *CEA ± sd* |
|---|---|---|---|
| Hep 2[a] | tumour $ng\,g^{-1}$ | 3558 ± 2689 | < 100 |
| | liver $ng\,g^{-1}$ | < 20 | < 20 |
| | blood $ng\,ml^{-1}$ | < 20 | < 20 |
| LS 174T | tumour $ng\,g^{-1}$ | < 100 | 51278 ± 31820 |
| | blood $ng\,ml^{-1}$ | < 20 | 51 ± 42 |
| RD | tumour $ng\,g^{-1}$ | < 100 | < 100 |
| | blood $ng\,ml^{-1}$ | < 20 | < 20 |
| Hep 2 TC[b] ng $10^{-7}$ cells | — | 2800[c] | < 20 |
| LS 174T TC ng $10^{-7}$ cells | — | not done | 5854 |

[a]After transplantation in nude mice.
[b]Tissue cultured cells.
[c]Since the weight of $10^7$ Hep 2 cells is ~0.05 g, the PLAP content of Hep 2 in TC, wet weight, is ~56 $\mu g\,g^{-1}$.

Hep 2 tumours taken from sacrificed animals contained PLAP (mean 3110 $ng\,g^{-1}$), but only background levels of CEA. There was a positive correlation between PLAP concentration in the tumours and amount of labelled antibody localized to the tumour ($r=0.66$, Table IV), but between tumour weight and concentration ratio the relation was inverse ($r=-0.50$). The results of immunofluorescence studies are shown in Table V. All Hep 2 cells in culture showed positive staining for PLAP. After they have been growing in nude mice and imprints were made, 40–50% of the cells were stained with polyclonal or monoclonal anti-PLAP. The mean intensity of stained cells was higher with polyclonal anti-PLAP antibodies than with the $F_{11}$ monoclonal antibody. This, however, is most likely a question of concentration of the active IgG in the antiserum, since rabbit anti-PLAP when further diluted showed the membrane staining pattern characteristic for $F_{11}$ (Figure 2). Monoclonal anti-PLAP blocked 50% of the polyclonal anti-PLAP fluorescence intensity/cell. Polyclonal anti-PLAP inhibited the staining with monoclonal anti-PLAP to <5%. Anti-CEA or anti-distemper did not inhibit anti-PLAP staining of Hep 2.

Monoclonal anti-distemper virus or normal mouse Ig did not stain *in vitro* grown Hep 2, Hep 2 imprints or RD. Anti-CEA stained tissue-cultured LS 174T and Detroit cells, but not Hep 2 or RD (Table V).

### Discussion

The present study was designed to explore the potential use of placental alkaline phosphatase as a target for immunodetection. This may be beneficial in diagnosis and monitoring of treatment in patients with seminoma (Wahren *et al.*, 1979; Lange *et al.*, 1982; Jeppsson *et al.*, 1983). Our results show that both polyclonal and monoclonal antibodies against PLAP localize to PLAP-producing tumours but not to tumours in which PLAP was not detectable. Evidence for the specific localization was the *in vitro* immunofluorescence of membrane and cytoplasm of the same type of tumour cells as those used *in vivo*. The *in vitro* localization could be blocked by polyclonal anti-PLAP and, as expected, partially blocked by monoclonal anti-PLAP, but was not blocked by control sera. The same type of blocking was not

**Table IV** Relation between tumour weight, concentration ratio and PLAP concentration in Hep 2 and LS 174T tumours

| | *Tumour weight in g, range* | *Concentration ratio of $^{125}I$ anti-PLAP, mean (range)* | *PLAP concentration $ng\,g^{-1}$, mean (range)* | *CEA concentration $ng\,g^{-1}$, mean (range)* |
|---|---|---|---|---|
| Hep 2 | 0.142–0.455 | 6.2 (2.6–10.5) | 3110 (500–9800) | 66 (39–98) |
| LS 174T | 0.100 | 0.6 | < 70 | 31836 |

**Table V** Mean fluorescence values and percent tumour cells stained with anti-PLAP and anti-CEA antibody

| | *F values with immunofluorescence* ± *sd* (*% stained cells*) | | | | | |
|---|---|---|---|---|---|---|
| | *Rabbit anti-PLAP*[a] | | $F_{11}$ | | *Rabbit anti-CEA* | |
| Hep 2 imprint | 46±7 | (40%) | 15±5 | (50%) | <5 | (0%) |
| Hep 2 TC[b] | 115±29 | (100%) | 20±3 | (100%) | 7±2 | (9%) |
| LS 174T TC | <5 | (0%) | <5 | (0%) | nm[c] | (50%) |
| Detroit 562 TC | <5 | (0%) | <5 | (0%) | 77±22 | (25%) |
| RD TC | <5 | (0%) | <5 | (0%) | <5 | (0%) |

[a]All antibody preparations were diluted 1:5 for staining, all preparations were air-dried.
[b]Tissue cultured cells.
[c]nm = not measured due to overlapping cell growth.

**Figure 2** Immunofluorescence photograph of Hep 2 cells grown in culture and stained with rabbit anti-PLAP (a, intracytoplasmic and cell membrane staining) and monoclonal anti-PLAP (b, cell membrane staining). ×576.

attempted *in vivo,* but it is unlikely that complete blocking should occur in the comparatively large tumours carried by the nude mice.

The amounts of labelled antibody bound to the tumour showed a positive although not very high correlation with the PLAP concentration. Smaller tumours had somewhat higher mean concentration ratios than larger tumours. This would be an advantage if the method was to be used for diagnostic purposes. The control anti-CEA antibodies gave variable binding ratios to Hep 2. The reasons are not clear, since the immunofluorescence studies and RIA determinations show very low CEA content in Hep 2 cells.

Both the rabbit polyclonal antibodies and the $F_{11}$ monoclonal antibody against PLAP were successful in localizing the tumour as compared to other tissues. The concentration ratios of blood however, were as high as for the tumour. This was previously seen also with anti-CEA injected to mice with CEA-containing tumours (Mach *et al.,* 1974; Hedin *et al.,* 1982). The blood concentration ratio thereafter declined, giving better tumour localization. The high initial concentration ratio in blood could represent circulating radiolabelled anti-PLAP, partially broken-down labelled antibody and perhaps free iodine from the broken-down injected material. There is probably not much binding of antibody to circulating antigen, since the PLAP concentration in blood of the nude mice was non-measurable. In future it would probably be an advantage to use fragments of antibodies for immunolocalization, since these are excreted quicker than whole IgG.

While 100% Hep 2 cells grown *in vitro* stained with high intensity for PLAP antibodies, the percentage and intensity decreased in the solid tumours after *in vivo* growth. Other authors have recently demonstrated that different subclones of HeLa cells expressing PLAP *in vitro*, when grown *in vivo* undergo modulation of their alkaline phosphatase pattern, by expressing intestinal type or tissue-unspecific type alkaline phosphatase at the expense of PLAP expression (Singer *et al.*, 1980). It is conceivable that the percentage of cells that still express PLAP are also undergoing modulation and thus contain a lower total amount. In tissue-cultured Hep 2 cells, the mean amount of PLAP appeared to be higher than in cells passed in the nude mice.

PLAP is produced by a highly polymorphic gene locus that includes more than 18 allelic forms as described by electrophoresis. Normally PLAP is expressed in second and third trimester placentae, but also by normal endocervix. The Hep 2 cells that were used for our experiments are subcloned from HeLa cells derived from a cervix carcinoma. HeLa cells have been shown to express the homozygote type 1 (old nomenclature type S) allelic forms of PLAP (Beckman *et al.*, 1970). The $F_{11}$ monoclonal antibody used in the present study reacts with the type 1 form of PLAP but shows restricted specificity since it does not react with the type 2 allelic form of PLAP (Millán *et al.*, 1982). In contrast, tumours like seminoma and different ovarian carcinomas express a PLAP-like enzyme (Wahren *et al.*, 1979; Benham *et al.*, 1981) that although cross-reactive with polyclonal antibodies to PLAP, can be distinguished from the normal placental phenotypes by use of different monoclonal antibodies (Millán *et al.*, unpublished results). A high percentage of these tumour enzymes is indeed non-reactive with the $F_{11}$ monoclonal antibody (Millán *et al.*, 1982). Thus polyclonal antibodies or monoclonal antibodies with specificities to common determinants should be used to allow radioimmunodetection of seminomas.

This study was supported by grants from the Swedish Cancer Society, pr 1387, Cancerföreningen, Stockholm, and Umeå University. We thank Agneta von Gegerfelt for skilful technical assistance, Susanne Kellquist and Marianne Lundberg for expert secretarial help.

**References**

BECKMAN, G., BECKMAN, L. & LUNDGREN, E. (1970). Isoenzyme variations in human cells grown *in vitro*. IV. Identity between alkaline phosphatases from HeLa cells and placenta? *Hum. Hered.*, **20**, 182.

BENHAM, F. J., FOGH, J. & HARRIS, H. (1981) Alkaline phosphatase expression in human cell lines derived from various malignancies. *Int. J. Cancer*, **27**, 637.

CHANG, C.H., ANGELLIS, D. & FISHMAN, W.H. (1980). Presence of the rare D variant heat-stable, placental type alkaline phosphatase in normal human testis. *Cancer Res.*, **40**, 1506.

FISHMAN, W.H., INGLIS, N.I., STOLBACH, L.L. & KRANT, M.S. (1968). A serum alkaline phosphatase isoenzyme of human neoplastic cell origin. *Cancer Res.*, **28**, 150.

FISHMAN, W.H. & STOLBACH, L.L. (1979). Placental alkaline phosphatase. In *Immunodiagnosis of Cancer*, (Eds. Herberman & McIntire) New York: Marcel Dekker Inc, p. 442.

GOLDSTEIN, D.J., BLASCO, L. & HARRIS, H. (1980). Placental alkaline phosphatase in nonmalignant human cervix. *Proc. Natl Acad. Sci.*, **77**, 4226.

HEDIN, A., WAHREN, B. & HAMMARSTRÖM, S. (1982). Tumor localization of CEA-containing human tumors in nude mice by means of monoclonal anti-CEA antibodies. *Int. J. Cancer*, **30**, 547.

HOLMGREN, P.-Å., STIGBRAND, T., DAMBER, M. & VON SCHOULTZ, B. (1978). A double antibody solid phase radioimmunoassay for placental alkaline phosphatase. *Clin. Chim. Acta*, **83**, 205.

JAVADPOUR, N. (1983). Multiple biochemical tumor markers in seminoma. *Cancer*, **52**, 887.

JEPPSSON, A., WAHREN, B., STIGBRAND, T., EDSMYR, F. & ANDERSSON, L. (1983). A clinical evaluation of serum placental alkaline phosphatase (PLAP) in seminoma patients. *Br. J. Urol.*, **55**, 73.

LANGE, P.H., MILLÁN, J.L., STIGBRAND, T., VESELLA, R.L., RUOSLAHTI, E. & FISHMAN, W.H. (1982). Placental alkaline phosphatase as a tumor marker for seminoma. *Cancer Res.*, **42**, 3244.

MACH, J.P., CARREL, S., MERANDA, C., SORDAT, B. & CERROTTINI, J.C. (1974). *In vivo* localization of radiolabelled antibodies to carcinoembryonic antigen in human colon carcinoma grafted into nude mice. *Nature*, **248**, 704.

MILLÁN, J.L., STIGBRAND, T., RUOSLAHTI, E. & FISHMAN, W.H. (1982). Characterization and use of an allotype-specific monoclonal antibody to placental alkaline phosphatase in the study of cancer-related phosphatase polymorphism. *Cancer Res.*, **42**, 2444.

SINGER, R.M., LEAHY, E.M. & GERSHWIN, M.E. (1980). Fetal isoenzyme expression of heterotransplanted HeLa cells in nude mice. *Exp. Cell Biol.*, **48**, 298.

WAHREN, B., HOLMGREN, P.-Å. & STIGBRAND, T. (1979). Placental alkaline phosphatase, alphafetoprotein and carcinoembryonic antigen in testicular tumors. Tissue typing by means of cytological smears. *Int. J. Cancer*, **24**, 749.

ZIMMERMAN, R. (1979). Improved performance of a double antibody radioimmunoassay for carcinoembryonic antigen. *J. Immunol. Meth.*, **25**, 311.